# GDF11 alleviates secondary brain injury after intracerebral hemorrhage via attenuating mitochondrial dynamic abnormality and dysfunction

**DOI:** 10.1038/s41598-021-83545-x

**Published:** 2021-02-17

**Authors:** Anqi Xiao, Yiqi Zhang, Yanming Ren, Ruiqi Chen, Tao Li, Chao You, Xueqi Gan

**Affiliations:** 1 Department of Neurosurgery, West China Hospital, Sichuan University, Chengdu, 610041 Sichuan People’s Republic of China; 2 Clinical Medicine, West China Medical Center of Sichuan University, Sichuan University, Chengdu, 610041 Sichuan People’s Republic of China; 3 West China–Washington Mitochondria and Metabolism Center and Department of Anesthesiology, West China Hospital, Sichuan University, Chengdu, 610041 Sichuan People’s Republic of China; 4 State Key Laboratory of Oral Diseases, West China Hospital of Stomatology, Sichuan University, 14 S Renmin Rd. 3rd Sec., Chengdu, 610041 Sichuan People’s Republic of China

**Keywords:** Neuroscience, Cell death in the nervous system, Experimental models of disease

## Abstract

Intracerebral hemorrhage (ICH) is a serious public health problem with high rates of death and disability. The neuroprotective effect of Growth Differentiation Factor 11 (GDF11) in ICH has been initially proved by our previous study. Oxidative stress (OS) plays crucial roles in mediating subsequent damage of ICH. However, whether and how mitochondrial dynamic events and function participated in ICH pathophysiology, and how mitochondrial function and OS interreacted in the neuroprotective process of GDF11 in ICH remains unclarified. Based on the rat model of ICH and in vitro cell model, we demonstrated that GDF11 could alleviate ICH induced neurological deficits, brain edema, OS status, neuronal apoptosis and inflammatory reaction. In addition, mitochondrial functional and structural impairments were obviously restored by GDF11. Treatment with antioxidant protected against erythrocyte homogenate (EH) induced cell injury by restoring OS status and mitochondrial fusion fission imbalance, which was similar to the effect of GDF11 treatment. Further, inhibition of mitochondrial division with Mdivi-1 attenuated mitochondrial functional defects and neuronal damages. In conclusion, our results for the first time proposed that GDF11 protected the post-ICH secondary injury by suppressing the feedback loop between mitochondrial ROS production and mitochondrial dynamic alteration, resulting in attenuated mitochondrial function and amelioration of neural damage.

## Introduction

Intracerebral hemorrhage (ICH) is a serious public health problem with high rates of death and disability, and it accounts for 20–30% of stroke cases in Asia^1,^^2^. The pathophysiology of intracerebral hemorrhage (ICH) damage is complicated. There is general agreement that the acute formation of the hematoma produces tissue disruption and displacement^3^. The intra-parenchymal hematoma triggers a series of events termed secondary injury, resulting progression of neurological deficits or death^4,5^. Of note, oxidative stress (OS) plays a key role in the pathogenesis of secondary ICH injury^6^. The production and accumulation of reactive oxygen species (ROS) mainly come from the accumulation of hemoglobin cleavage products, excitatory amino acids and inflammatory factors, and excessive generation of ROS triggers protein damage, neuronal apoptosis, and neuro-inflammation, which lead to neurological deterioration in ICH patients^3,7,8^.

Mitochondria, the unique organelles for generating energy, are also the immediate target and main intracellular source of ROS^9^. Mitochondrial dysfunction caused the dysregulation of ROS homeostasis, ROS accumulation further caused damage in mitochondria, and persistence of vicious circles^10^. Previous studies presented evidences that links mitochondrial impairment to ICH. The initial hematoma after ICH induces glutamate release and then leads to mitochondrial dysfunction of neurons. Sook Kim-Han et al. found abnormal mitochondrial respiration function as early as 2 h after ICH and a progressive decline with longer periods after ICH^11^. Extensive mitochondrial vacuolization and mitochondria swelling were also presented in brain tissue of ICH diabetic rats^12^. Mitochondrial dysfunction may contribute to the deficiency of adenosine triphosphate (ATP) generation and then result in further excessive ROS generation and neuronal apoptosis and aggravation of brain injury after ICH^12,13^. Hence, oxidative stress status and mitochondrial disorders are deeply involved in secondary ICH injury, management of oxidative stress and mitochondrial dysfunction may be an effective therapeutic strategy for brain injury after ICH. However, the underlying mechanisms are not well understood and specific evidences remain scarce.

As a dynamic organelle, mitochondrion undergoes fission and fusion continuously^14^. The health of mitochondrial dynamics (fission and fusion) plays a critical role on maintaining mitochondrial morphology, appropriate distribution and normal function^15,16^. The balance of fusion and fission relays on the regulation of fusion related proteins, like dynamin-related GTPases mitofusin 1 and 2 (Mfn1 and Mfn2), [optic atrophy1 (OPA1)] and fission related proteins, like dynamin-related protein 1 (Drp1), Fission 1 (Fis1)^17,18^. For mitochondrial dynamic properties are essential for neuronal activity^19^, imbalance of fusion and fission could result in aberrant distribution of mitochondria and defective cellular function^20,21^. Sufficient evidence demonstrated that mitochondrial dynamics are highly related to mitochondrial electron transport chain (ETC) function, mitochondrial ROS (mtROS) accumulation, and ATP production in many neurogenerative diseases, such as Alzheimer’s Disease, Parkinson’s, multiple sclerosis, and so on^22^. However, hitherto, whether and how mitochondrial dynamic balance involves in ICH pathophysiology remains unexplored.

Growth Differentiation Factor 11 (GDF11), a member of TGF-b super-family, is considered as a “rejuvenate factor” in improving diseases of different systems on old individuals^3,9^. GDF11 acts through the activin type II receptor (ActRIIB), and are antagonized by the activin-binding protein follistatin^23,24^. Number of studies proved that GDF11′s binding of activin to ActRIIB induces the recruitment and phosphorylation of an activin type I receptor, which then phosphorylates the intracellular signalling proteins SMAD2 and SMAD3^25^, consequently affecting cell function and differentiation, reverse the aging and degradation of the nervous central system(CNS), myocardium and skeletal muscle^26–28^. Previous studies on myocardial ischaemia/reperfusion injury also demonstrated that GDF11 administration increased the phosphorylation of SMAD3 and decreased the phosphorylation of FOXO3a, which then affected mitochondrial Ca^2+^ homeostasis, ROS accumulation and mitochondrial depolarization^29^. GDF11 also exerted its neuroprotective effect through the ALK5-Smad2/3 pathway in cerebral ischemic injury to reduce cell apoptosis of neurons in the peri-infarct cerebral cortex^30^. Moreover, researchers revealed the anti-OS effect of GDF11 by alleviating the accumulation of ROS and its subsequent damage in cardio/cerebrovascular diseases^31^. In our previous study, the preliminary neuroprotective role of GDF11 in ICH has been explored^1^. However, its regulation of neuro-mitochondria and specific antioxidant stress effect against secondary ICH injury is undocumented.

Therefore, the aims of the present in vivo and in vitro study were to investigate (1) whether and how mtROS mediated mitochondrial dysfunction were involved in the neuroprotective regulation of GDF11 in ICH; (2) whether and how mitochondrial dynamic events participated in the neuroprotective role of GDF11 in ICH; (3) the interreaction and relationship between mitochondrial dynamic abnormality and mtROS accumulation in the neuroprotective process of GDF11 in ICH. Our results would provide new insight into the underlying mechanisms of GDF11 neuroprotection on ICH pathogenesis, highlighting the potential therapeutic application for ICH.

## Material and methods

### Animal preparation, set up ICH model and neurological function investigation

All experimental procedures were approved by Biological and Medical Ethics Committee of West China Hospital, Sichuan University and complied with the Guide for the Care and Use of Laboratory Animals by National Institutes of Health. According to our previous study, we detected that serum GDF11 concentrations were significantly lower in aged rats^1^. Thus, 45 aged Male Sprague–Dawley (SD) rats (24 months old; 320–360 g) were used (Dashuo Laboratory Animal Co., Ltd, Chengdu, China). All animals were housed in an environmental controlled room with 12 h light/dark cycle under fixed temperature (21 °C) and allowed free access to food and water. In addition, their health and weight condition were monitored daily. 45 rats were randomly and equally divided into three groups: sham + vehicle group, ICH + vehicle group, and ICH + rGDF11 group (n = 15 for each group). The GDF11 stock solution was diluted to 0.1 mg/mL with 30 mM acetate buffer for intraperitoneal (IP) injections. IP injections of recombinant GDF11 (R&D Systems, catalog No.1958-GD-010) (0.1 mg/kg/day) or vehicle only (30 mM acetate) were first given 2 h prior to operation and daily continued until the end of this experiment (7 days after operation).

The ICH model was set as previously described^1^. In brief, rats were anesthetized intraperitoneally with 4% chloral hydrate. Then specimens were set in a stereotactic apparatus frame (RWD, Shanghai, China) and made an incision about 1.5 cm over the anterior scalp. The right basal ganglia was stereotactically localized as: 3.5 mm lateral to the midline, 1.5 mm posterior to the bregma, and 5.5 mm ventral to the cortical surface. After drilling a 1-mm-diameter burr hole in the skull, autologous whole blood (80 μL) taken from central tail artery was injected into the right basal ganglia at a constant rate of 16 μL/minute via microinfusion pump. After that, needle was stayed for extra 10 min and then withdrawn slowly. At last, sealed the burr hole by bone wax and sutured the scalp. During the procedures, the body temperature was maintained at 37 + 0.5 °C using a feedback-controlled heating blanket. Behavioral testing was performed on day 1, day 3 and day 7 after ICH in all experimental groups as our previous study did. Modified neurological stroke scale (mNSS) was used to evaluate rat’s behavior among groups in terms of motor, sensory, balance beam tests, and the presence of reflexes or abnormal movements. For brain tissue testing, the intact rat brain was removed after anesthetic sacrifice with 4% chloral hydrate intraperitoneal injection. Evaluation of the brain water content, immunohistochemistry staining of OX-42, and cell apoptosis evaluation by TUNEL staining were all performed as previously described^1^.

### Oxidative stress level measurement

ICH hemispheres were collected from each group and homogenized and centrifuged at 4 °C^32^. The concentrations of glutathione (GSH), superoxide dismutase (SOD), and malondialdehyde (MDA) in the homogenate were determined by commercial kits according to the supplier’s instructions (Nanjing Jiancheng Bioengineering Institute, Nanjing, China)^33^. The activity of glutathione peroxides (GPx) and activity of glutathione-*S*-transferase (GST) were measured from brain tissue homogenate according to the instruction of commercial kits (Nanjing Jiancheng, Bioengineering Institute, Nanjing, China)^34,35^.

### Measurement of enzyme activities associated with respiratory chain complexes and ATP levels

Mitochondrial respiration complex activity was measured in ICH hemispheres or in SH-SY5Y cell homogenates as described before^36,37^. Briefly, ICH hemispheres or cultured cells were homogenized and sonicated in the isolation buffer containing 250 mM sucrose, 20 mM HEPES, pH 7.2, and 1 mM EDTA. Complex I (NADH-ubiquinone reductase) activity was determined in 25 mM potassium buffer containing KCl, Tris–HCl and EDTA (pH 7.4). The change in absorbance was monitored at 340 nm wavelength every 20 s for 6 min using an Amersham Biosciences Ultrospect 3100 pro spectrophotometer. For homogenized samples (50 µg protein), the oxidation of NADH was recorded for 3 min following the addition of 2 µg/mL antimycin, 5 mM MgCl_2_, 2 mM KCN, and 65 µM co-enzymes Q1 to the assay mixture, and then 2 µg/mL rotenone was added to the mixture. The absorbance of samples was measured for another 3 min. Enzyme activities in complex II (succinate dehydrogenase), complex III (ubiquinol-cytochrome c reductase), complex IV (cytochrome c oxidase, CcO), and citrate synthase were determined as described previously^38^. ATP levels were measured using an ATP Bioluminescence Assay Kit (Roche) following the manufacturer’s instructions^39^. ICH hemispheres and SH-SY5Y cells were homogenized in the lysis buffer provided in the kit, incubated on ice for 15 min, and centrifuged at 14,000*g* for 15minutes. Subsequent supernatants were measured for the ATP levels using Luminescence plate reader (Molecular Devices) with an integration time of 10 s.

### Cell culture and treatments

The SH-SY5Y human neuroblastoma cells (obtained from ATCC) were cultured as monolayer in polystyrene dishes and maintained in DMEM supplemented with 2 Mm glutamine, 100 units/mL penicillin, 100 mg/mL streptomycin, and 10% of FBS at 37 °C in a humidified atmosphere of 5% CO_2_. Cells were subcultured at confluences (70–80%), and the medium was replaced twice a week. For ICH-like injury model, we exposed SH-SY5Y human neuroblastoma cell cultures to freshly prepared erythrocyte homogenate (EH). The RBC isolated using density gradient centrifugation (BD Vacutainer CPT) were lysed in distilled H2O at a density of 1 × 10^9^ RBC/mL, and the RBC lysate was added into the culture medium to the hemoglobin (Hb) concentration of 0.1, 1, 5, 10 m$\upmu$, measured by spectrophotometer as previous study described^7^. For EH treated group, SH-SY5Y cells were treated with EH, which contained Hb (0.1, 1, 5, 10 m$\upmu$) for 24hs, and for EH + GDF11 group, recombinant human GDF 11 (R&D Systems) with 10 ng/mL working concentration was added 12hs prior to the EH treatment^40^. The antioxidant N-acetylcysteine (NAC) (working concentration: 1.0 mM; Sigma Aldrich Co., St Louis, MO, USA) and mitochondrial division inhibitor 1 (Mdivi-1) (working concentration: 25 μM; Sigma Aldrich Co., St Louis, MO, USA) were dissolved in DMSO (50 mM) and diluted with culture medium to the working concentration. NAC or Mdivi-1 was co-added with EH to culture medium for EH + NAC and EH + Mdivi-1 groups.

### Determination of LDH

Lactate dehydrogenase (LDH) is an enzyme that catalyzes the conversion of lactate to pyruvate, which is an important step in energy production in cells. The cell injury was assessed by determining the amount of LDH released into the culture medium, using an LDH assay kit (Promega)^41^.

### Measurement of apoptosis by flow cytometry and TUNEL assays

Measurement of apoptosis by flow cytometry was performed as previously described^42^. An annexin V-fluorescein isothiocyanate (FITC) Apoptosis Detection Kit (annexin V-FITC conjugate, propidium iodide (PI) dyes, and binding buffer) (BD Pharmingen, California, USA) was used. Cells were washed with PBS and dissolved in binding buffer. Annexin-V was added and cells were incubated in the dark for 15 min. Subsequently, propidium iodide (PI 1 μg/mL) was added. After exposure to various experimental conditions, cells were labeled with fluorochromes at 37 °C after trypsinization. Then cytofluorometric analysis was performed with FACScanto II flow cytometer (Becton Dickinson, Mountain View, CA, USA).

TUNEL staining was performed to evaluate the cell apoptosis in brain tissues or in SH-SY5Y cells as previously described^43^. The extent of cell death was assessed using a Terminal deoxynucleotidyl Transferase Biotin-dUTP Nick End Labeling (TUNEL) kit (Roche), according to the manufacturer’s instruction. In brief, fixed slides were pretreated with 20 mg/mL proteinase-K in 10 mM Tris–HCl at 37 °C for 15 min and then incubated in 0.3% hydrogen peroxide dissolved in anhydrous methanol for 10 min after being rinsed in phosphate-buffered saline (PBS), then incubated in 0.1% sodium citrate and 0.1% Triton X-100 solution for 2 min at 4 °C. After PBS washes, samples were incubated with 50 μL of TUNEL reaction mixture with terminal deoxynucleotidyl transferase (TdT) at 37 °C for 1 h, and the neuronal nuclei were stained with 40,6-diamidino-2-pheny lindole (DAPI). Sections were visualized by a fluorescence microscope (Leica TCS SPE, Germany). Images were acquired from 4 randomly selected fields (40×) at both the edge and center of the hematoma region. The total number of nuclei and TUNEL positive cells were counted in each field of view.

### Western blot analyses

Expression levels of target proteins were measured by western blot as previously described^44^. After the indicated treatments, cells were collected and lysed in cell lysis buffer (Cell Signaling Technology, Beverly, MA, USA). Protein concentrations were determined using a Bradford protein assay kit (Thermo Fisher Scientific). Proteins were electrophoresis by SDS-PAGE and transferred to a polyvinylidene difluoride (PVDF) membrane. Anti-Drp1 (1:1000, BD Science), anti-phospho-Drp1(Ser616) (1:1000, Cell Signaling), anti-Opa1 (1:2000, BD Science), anti-Mitofusion2 (Mfn2) (1:1000, Abcam), anti-Fis1 (1:1000, Atlas Antibodies), anti-VDAC (1:2000, Santa Cruz), anti-Bcl-2 (1:1000, Santa Cruz), anti-Bax (1:2000, Santa Cruz), anti-Caspase-3 (1:1000, Santa Cruz), anti-Cleaved Caspase-3 (1:1000, Santa Cruz), and anti-β-actin (1:8000, Sigma) antibodies were used as primary antibodies. The binding sites of primary antibody were visualized with horseradish peroxidase-conjugated anti-rabbit IgG antibody (1:5000, Invitrogen) or anti-mouse IgG antibody (1:5000, Invitrogen), followed by the addition of ECL substrate. The protein bands were detected using a Bio-Rad imaging system (Bio-Rad, Hercules, CA, USA) and quantified with NIH ImageJ software (available in the public domain) and normalized with b-actin levels.

### Functional imaging assays

Cells were harvested from 75 cm^2^ flasks and replated at low density onto Lab-Tek eight-well chamber slides. Intracellular ROS was detected by DCF staining with 2′,7′-dichlorodihydrofluorescein diacetate (H2DCF‐DA) (10 μM; Sigma) for 30 min. Mitochondrial ROS generation was determined by co-staining with Mitotracker Green (MTGreen) (100 nM; Molecular Probes) and Mitosox Red (2.5 μM; Molecular Probes) (a unique fluorogenic dye highly selective for detection of superoxide production in live cell mitochondria) for 30 min. For mitochondrial membrane potential determination, cells were co-stained with MTGreen (100 nM; Molecular Probes) and Tetramethylrhodamine methyl ester (TMRM) (100 nM; Molecular Probes) for 30 min. Fluorescence from MTGreen is independent of membrane potential, whereas TMRM is sensitive to membrane potential. Mitochondria were labeled with Mitotracker Red (Molecular Probes, incubated in 100 nM Mitotracker Red for 30 min at 37 °C before fixation) to visualize morphology. Cells were transfected with 1 µg mito-DsRed plasmids (pDsRed2-Mito). 24 h after transfection, pDsRed2-Mito positive cells were recorded.

Images were captured under a microscope (Leica TCS SPE). Excitation wavelengths were 543 nm for Mitosox, TMRM, Mitotracker Red or pDsRed2-Mito and 488 nm for MTGreen, respectively. Fluorescent signals were quantified using NIH Image J software. Post-acquisition processing was performed with MetaMorph (Molecular Devices) and NIH Image J software for quantification and measurement of fluorescent signals of mitochondrial length and density. Mitochondrial size, shape, density, and fluorescent intensity were quantified by an investigator blinded to experimental groups. More than 100 clearly identifiable mitochondria from randomly selected 10–15 cells per experiment were measured in 3 independent experiments as our previous study described^45^.

### Statistical analysis

Data were statistically analyzed using Prism software for Student’s t test and are presented as mean ± standard deviation (SD). The statistical comparisons among multiple groups were made using one-way ANOVA, and multiple time points by two way ANOVA followed by Bonferroni correction. In all instances, N refers to the number of animals in a particular group. A p value of < 0.05 is considered statistically significant.

## Results

### GDF 11 attenuated ICH secondary injuries in elderly rats

**Figure 1:**
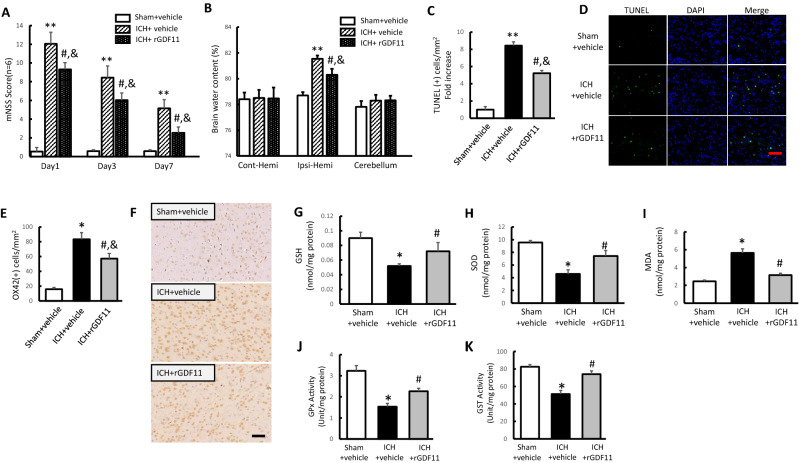
GDF 11 attenuated ICH secondary injuries in elderly rats. (**A**) Modified neurological severity scores (mNSS) of different groups on day 1, 3, and 7 post-ICH. (**B**) Brain water content of contralateral hemisphere, ipsilateral hemisphere and cerebellum on day 3 post-ICH expressed as percentage of the wet weight. (**C**,**D**) The peri-hematomal TUNEL-positive cells on day 3 post-ICH, presenting as fold increases (**C**) and TUNEL/DAPI-fluorescence staining (scale bar 200 μm) (**D**). (**E**,**F**) OX-42 (+) cells counting (**E**) and OX-42 immunohistochemical staining (scale bar 50 μm) on day 3 post-ICH (**F**). (**G**–**I**) ICH-induced oxidative stress indicators shown as GSH (**G**), SOD (**H**) and MDA (**I**) on day 3 after ICH surgery. (**J**,**K**) GPx activity and GST activity in the indicated groups on day 3 after ICH surgery. Values shown as mean ± SD. *p < 0.05 and **p < 0.01 versus sham + vehicle group; ^#^p < 0.05 versus ICH + vehicle group and ^&^p < 0.05 versus sham + vehicle group. n = 6–8 animals per group. *TUNEL* terminal deoxynucleotidyl transferase dUTP nick end-labelling, *DAPI* 40,6-diamidino-2-phenylindole, *GSH* glutathione, *SOD* superoxide dismutase, *MDA* malondialdehyde.

Firstly, neurological behavioral testing in each group was evaluated before sacrifice. Compared to the sham group, severe behavioral impairment appeared in ICH groups on 1, 3, 7 days. While compared to ICH treated group (ICH + vehicle group) at each time point, rGDF11 treatment restored the neurological impairment after ICH. (Fig. 1A). To assess ICH-induced brain edema, we examined brain water content of each group on day 3 post-ICH. As shown in Fig. 1B, no significant difference was found in water content of contralateral hemisphere and cerebellum among the groups. However, compare to the sham, brain water content of ipsilateral hemisphere was found significantly increased in two ICH groups. While with further comparison in between, remarkable reduce of water content was presented in ICH + rGDF11 rats, indicating the ameliorating effect of GDF11 in ICH-induced edema (Supplementary Information S1).

The increase of neuronal apoptosis and inflammatory activity is a typical indicator for ICH secondary injury. Thus, we utilized TUNEL staining to evaluate peri-hematomal apoptosis on day 3 post-ICH induction. As shown in Fig. 1 C,D, compared with the Sham + vehicle group, number of TUNEL-positive cells in the peri-hematomal area increased as much as 8.43 times in ICH + vehicle group. While after rGDF11 treatment, a significant reduction was shown on TUNEL-positive cells, dropping by 46.11%. To evaluate inflammatory injury of the brain tissue, we examined OX-42^+^ microglial cells of each group on day 3 post-ICH. Based on the immunohistochemistry staining, OX-42^+^ microglial cells in ICH + vehicle rats increased significantly comparing to the Sham + vehicle group, while a clear inhibition was found in ICH rats with administration of rGDF11 (Fig. 1E,F).

Known as oxidative stress playing a crucial role in neuro-impairment after ICH, we tested the levels of GSH, SOD, MDA around the ipsilateral basal ganglia to explore the ICH-induced oxidative stress. After ICH, the obvious reduction of GSH and SOD levels and strong increase of MDA level was more pronounced in ICH + vehicle group as compared with the Sham + vehicle group, whereas after GDF11 treatment, these changes were significantly inhibited. Between Sham + vehicle and ICH + GDF11 groups, no significant differences were observed in indicated oxidative stress levels (Fig. 1G–I). GSH plays a role in myriad cellular functions, including maintenance of reduced protein thiols, detoxification of hydrogen peroxide (H_2_O_2_) and lipid peroxides, secondary metabolism, and non-enzymatic scavenging of free radicals. Intracellular GSH and its related enzymes, such as GPx and GST constitute the cellular glutathione antioxidant system and represent a crucial defensive system to protect cells against ROS. Based on that, we evaluated the activity of GPx and GST for different groups. As shown in Fig. 1J,K, GPx activity and GST activity in the ICH + vehicle group decreased significantly as compared with the Sham + vehicle group, whereas after GDF11 treatment, these changes were markedly restored. In this regard, our results indicated that GPx and GST activity increased by GDF11 enhanced the resistance to neuro-injury after ICH.

### GDF11 restored mitochondrial dysfunction in brain tissue in elderly ICH rats

**Figure 2:**
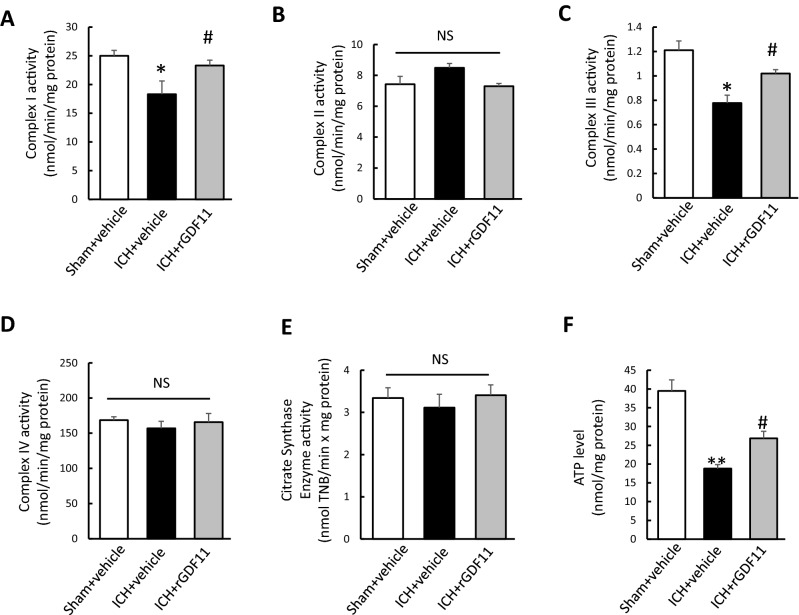
GDF11 restored mitochondrial dysfunction in brain tissue in elderly ICH rats. (**A**–**E**) Complex I, II, III, V and citrate synthase enzyme activities in the indicated groups on day 3 post-ICH. (**F**) ATP level reduction in indicated 3 groups. n = 6–8 animals per group. Values shown as mean ± SD. *p < 0.05 and **p < 0.01 versus sham + vehicle group; ^#^p < 0.05 versus ICH + vehicle group.

As mitochondrial functions are deeply involved in secondary ICH injury, we next evaluated mitochondrial function by measuring key enzyme activity associated with respiratory chain and adenosine triphosphate (ATP) levels. The activity of the key enzymes involved in ETC were evaluated on day 3 post-ICH. Regarding the activity of ETC enzymes, as shown in Fig. 2B,D, no significant difference was found in Complex II and Complex IV activities among three groups. However, the activities of Complex I and III were dramatically decreased in ICH + vehicle group as compared with Sham + vehicle (dropping by 73.2% and 64.2%, respectively, while GDF11 treatment significantly restored complex I and III activity (Fig. 2A–D). Citrate synthase activity, used as a quantitative enzyme marker for the presence of intact mitochondria, was comparable among three groups (Fig. 2E). Furtherly, we determined ATP levels on day 3 post-ICH using an ATP Bioluminescence Assay. The ATP level significantly decreased in ICH + vehicle group as compared with sham + vehicle group, however, a visible improvement on ATP levels was recorded in ICH rats with rGDF11 administration (Fig. 2F).

### GDF11 attenuated apoptosis and ROS accumulation of SH-SY5Y cells in ICH-like neurotoxicity model

**Figure 3:**
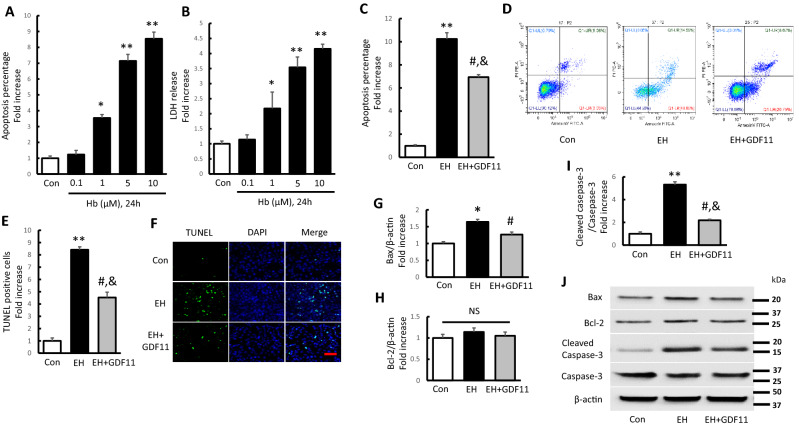
GDF11 attenuated EH-induced apoptosis and ROS accumulation in SH-SY5Y cells. (**A**,**B**) Quantification of apoptosis and cell released LDH in culture exposed to 0.1–10 μM Hb for 24 h. (Values shown as mean ± SD. *p < 0.05 and **p < 0.01 versus control group, n = 3). The cell cultures for EH treatment of the following experiments were exposed to 5 μM Hb environment for 24 h. (**C**,**D**) Bar graph and Flow cytometric quantification of apoptosis percentage presenting as fold increase (n = 7). (**E**,**F**) The TUNEL-positive cells presenting as fold increases and representative pictures for TUNEL/DAPI-fluorescence staining (scale bar 200 μm). (**G**-**J**) Quantification of immunoreactive bands for Bax (**G**), and Bcl-2 (**H**) relative to β-actin, and cleaved Caspase-3 (**I**) relative to Caspase-3. Immunoreactive bands for Bax, Bcl-2, cleaved Caspase-3, and Caspase-3 in the different group (**J**). Values shown as mean ± SD. *p < 0.05 and **p < 0.01 versus control group; ^#^p < 0.05 versus EH group and ^&^p < 0.05 versus control group (n = 7).

As there are no standard in vitro cell injury models that could mimic the ICH injury, we established the ICH-like in vitro model according to the previous study^7^. We exposed SH-SY5Y human neuroblastoma cell cultures to freshly prepared EH normalized by hemoglobin concentration. Hemoglobin (Hb) is the most abundant protein in RBCs. When released into the brain parenchyma during hemolysis, Hb becomes a central mediator of cytotoxicity^46,47^. Firstly, to evaluate cellular damage induced by EH with different dose of Hb concentration, we tested quantification of apoptosis percentage and cell released LDH in culture exposed to 0.1–10 μM Hb for 24 h. The result showed a dose-dependent increase in the apoptosis incidence and LDH release. EH containing 0.1 μM Hb treated cells showed comparable apoptosis percentage and LDH release with control group, while EH containing 1–10 μM Hb treated cells showed significant increase of apoptosis percentage and LDH release, and EH containing 5 μM Hb presented the ideal dose resulting in induced cellular damage (Fig. 3A,B).

Thus, we utilized the EH containing 5 μM Hb to establish ICH-like neurotoxicity model in following experiments. Subsequently, we used flow cytometric analysis and TUNEL/DAPI staining to explore the EH induced cell apoptosis (Fig. 3C–F). The result revealed a significant boost of apoptosis in both EH induced cohorts (EH and EH + GDF11 group), while compared with each other, the apoptosis was apparently reduced in EH + GDF11 group. For Bcl-2 family proteins play an important role in mediating apoptosis, we examined the expression changes of apoptosis-related proteins Bax and Bcl-2 in Hb-induced cells apoptosis by immunoblot analyses. Compared with the control group, EH increased the protein expression of Bax by 1.64 times, but did not markedly affect Bcl-2 expression (Fig. 3G,H). Furthermore, cleaved Caspase-3 relative to Caspase-3 was significantly enhanced by 5.32 times in EH treated cells (Fig. 3I). Notably, rGDF11 could apparently limit the expression of Bax and cleaved Caspase-3 in EH induced cells (EH + GDF11 group), reduced by 23.02% and 59.02%, respectively as compared with EH treated cells (EH group) (Fig. 3G–J). These results indicated that EH treatment obviously induced cell apoptosis, which could be evidently attenuated by rGDF11 treatment.

### GDF 11 attenuated mitochondrial dysfunction of SH-SY5Y cells in ICH-like neurotoxicity model

**Figure 4:**
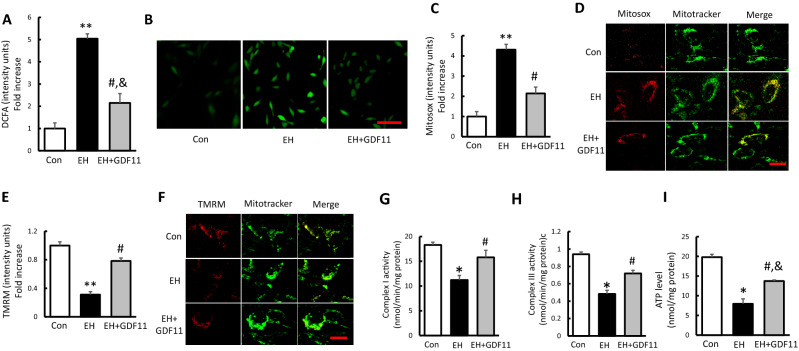
GDF 11 attenuated EH-induced mitochondrial dysfunction in SH-SY5Y cells. (**A**,**B**) Quantitative analysis of DCFA fluorescence intensity and representative images (scale bar 50 μm) in the indicated groups. (**C**,**D**) Quantification of Mitosox staining intensity and representative images showing Mitosox staining (scale bar 5 μm) in the indicated groups. (**E**,**F**) Quantification of TMRM staining intensity presenting as fold increase and representative images (scale bar 5 μm) in the indicated groups. (**G**–**I**) Complex I, III activities and ATP levels in the indicated groups. Values shown as mean ± SD. *p < 0.05 and **p < 0.01 versus control group; ^#^p < 0.05 versus EH group and ^&^p < 0.05 versus control group (n = 7).

ROS generates as normal byproducts of cellular metabolism. However, ROS production can increase dramatically under pathologic conditions, leading to cell damage. In this study, we first monitored the total cellular ROS formation in each group by DCFA staining. As shown in Fig. 4A,B, the level of the intracellular ROS significantly increased in EH treated cells, and decreased in EH + GDF11 group.

Given that mitochondria are a major source of ROS generation and that ROS accumulation affects mitochondrial function, we tested whether mitochondrial ROS generation correlates with mitochondrial dysfunction. Indeed, the intensity of Mitosox staining, an indicator for mitochondrial ROS, was significantly increased in EH treated cells by 4.31 times compared to control cells, and rGDF11 significantly suppressed mitochondrial ROS accumulation (Fig. 4C,D). To determine whether EH treatment altered mitochondrial functions in SH-SY5Y cells and the effect of GDF11 on EH induced mitochondrial dysfunction, the mitochondrial membrane potential (Δ*mΨm*), ETC activities and ATP production were evaluated in each group. As shown in Fig. 4E,F, TMRM staining was significantly decreased in EH treated cells by 63.5% compared to control group, and rGDF11 greatly restored that. Regarding the activity of ETC enzymes, the activities of Complex I and III were apparently decreased in EH treated cells compared with the control group (Fig. 4G,H). In consistency, great suppression of ATP level was also found in EH group (Fig. 4I). In EH + GDF11 group, ETC enzyme activity and ATP production were dramatically restored, indicating the protective potential of GDF11 against EH induced mitochondrial dysfunction (Fig. 4G–I). These results suggested that ICH-like treatment obviously induced mitochondrial dysfunction, which could be significantly restored by GDF11.

### ICH-like treatment altered mitochondrial dynamic abnormality, which could be restored by rGDF11 treatment

**Figure 5:**
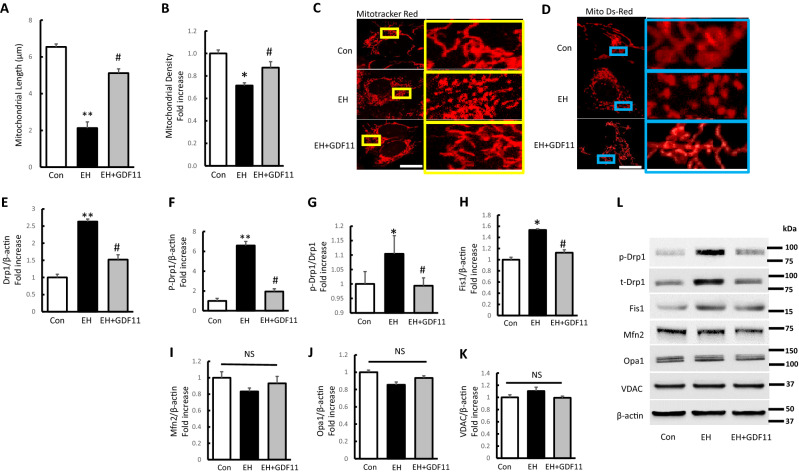
EH altered mitochondrial dynamic abnormality, which could be restored by rGDF11 treatment. (**A**–**D**) Quantification of mitochondrial length and density and representative images of Mitotracker Red and Mito Ds-Red staining (scale bar 5 μm) in the indicated groups. (**E**–**L**) Immunoreactive bands and quantification for p-Drp1 relative to Drp1(G) and Drp1, p-Drp1, Fis1, Mfn2, Opa1 and VDAC relative to β-actin in the indicated groups with DNA ladder markers on left side (L). Values shown as mean ± SD. *p < 0.05 and **p < 0.01 versus control group; ^#^p < 0.05 versus EH group (n = 7).

We further evaluated the effects of GDF11 on mitochondrial dynamic events in EH treated cells. Our result showed quantification of both mitochondrial length and density were significantly reduced by EH induction compared to control group (Fig. 5A,B). Morphologically, the mitochondria under EH induction were misshapen and fragmented indicated by specific Mitotracker Red staining and mito-DsRed transfection (Fig. 5C,D). In contrast, the mitochondria in the control group exhibited an elongated-tubular and filamentous morphology within an even distribution in cytoplasm. Abnormal mitochondrial morphology in EH treated cells (fragmentation) was largely reversed with rGDF11 treatment, the decrease of mitochondrial length and density was remarkably resisted associated with morphological improvement (Fig. 5A–D), indicating the protective effect of rGDF11 on mitochondrial dynamics.

Related fission and fusion proteins regulate the mitochondrial dynamics, maintaining mitochondrial morphology^48^. Drp1 is a key player in mitochondrial fission regulation co-acting with other proteins. Accumulating evidences suggest Drp1 is able to be phosphorylated at several sites, and phosphorylated Drp1 at the Ser616 site was proved to be related to the mitochondrial fragmentation^49^. Indeed, Drp1 levels were significantly increased in EH treated cells by 2.63 times as compared with control group, and we observed an increase in Ser616 phosphorylation in parallel with an increase in total Drp1 in EH treated cells as well as p-Drp1 (at 616 residues) contrast with total Drp1 protein levels (Fig. 5E–G,L). Fis1, another important regulator of mitochondrial fission, was also upregulated in Hb-treated cells by 1.74 times (Fig. 5H,L). However, rGDF11 treatment (EH + GDF11 group) could successfully suppress the increase of p-Drp1, Drp1 and Fis1 expression compared to EH treated group. While among the indicated groups, no significant difference was observed in the protein expression of the Mfn2, or Opa1 (Fig. 5I,J,L). As Voltage-dependent anion channel (VDAC) is involved in the regulation of mitochondrial physiology and is a critical component in controlling mitochondrial energy production^50^, we performed western blot for VDAC levels. The results showed that no differences were found in different groups (Fig. 5K,L). It suggested that VDAC did not play effect on the mitochondrial dysfunctions induced by EH. These data demonstrated that impaired mitochondrial fission and fusion dynamics were involved in the EH induced cell injury, and the protection role of rGDF11 was closely associated with its restoration effect on the altered mitochondrial dynamics.

### Effect of antioxidant treatment on erythrocyte homogenate induced apoptosis and mitochondrial dysfunction in SH-SY5Y cells

**Figure 6:**
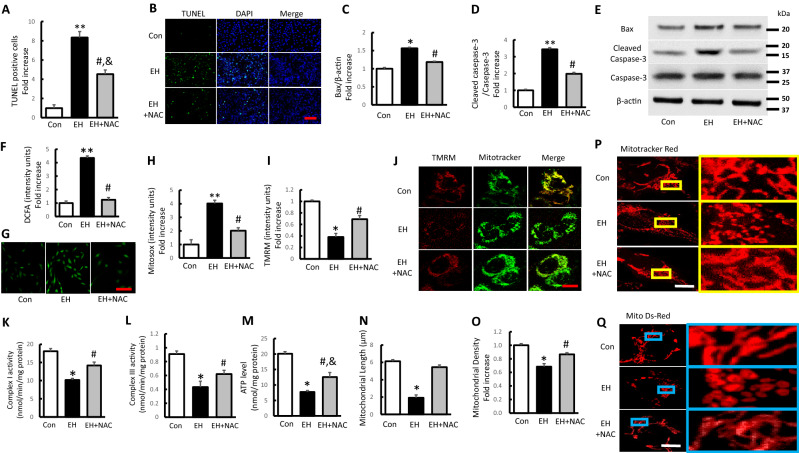
Effect of antioxidant treatment on EH-induced apoptosis and mitochondrial dysfunction in SH-SY5Y cells. (**A**,**B**) The TUNEL-positive cells presenting as fold increase and representative pictures for TUNEL/DAPI-fluorescence staining (scale bar 200 μm) in indicated groups. (**C**–**E**) Quantification of immunoreactive bands for Bax relative to β-actin (**C**) and cleaved Caspase-3 relative to Caspase-3 (**D**). Immunoreactive bands for Bax, cleaved Caspase-3, and Caspase-3 in the different treated cells (**E**). (**F**,**G**) Quantitative analysis of DCFA fluorescence intensity presenting as fold increase and representative images (scale bar 50 μm) in the indicated groups. (**H**–**J**) Quantification of Mitosox and TMRM staining intensity (**H**,**I**) with representative images showing TMRM staining (scale bar 5 μm) in the indicated groups (**J**). (**K**–**M**) Complex I, III activities and ATP levels in the indicated groups. (**N**–**Q**) Quantification of mitochondrial length and density (**N**,**O**) with representative images of Mitotracker Red and Mito Ds-red staining (scale bar 5 μm) in the indicated groups (**P**,**Q**). Values shown as mean ± SD. *p < 0.05 and **p < 0.01 versus control group; ^#^p < 0.05 versus EH group and ^&^p < 0.05 versus control group (n = 7).

In view of increased ROS production and accumulation in EH treated cells and a critical contributor of oxidative stress to mitochondrial dysfunction and abnormal changes in mitochondrial structure^51^, we next determined if antioxidant treatment could rescue altered EH induced cell damage, apoptosis, mitochondrial functional and structural impairment. Cells were incubated with the antioxidant NAC, which is a precursor of GSH. As the result, NAC treatment rescued cellular apoptosis in EH treated group, indicated by decreased TUNEL positive cells and depression of Bax and cleaved Caspase-3 (Fig. 6A–E). In terms of anti-ROS effect, NAC significantly ameliorated the intracellular and mitochondrial OS levels induced by EH induction, indicated by remarkable reduction of DCFA and Mitosox intensity (Fig. 6F–H). Such treatment significantly improved mitochondrial function and energy metabolism by increased membrane potential (as measured by TMRM staining), complex I and III activity, and ATP levels in EH treated cells (Fig. 4I–M). The protective effect of NAC on EH induced mitochondrial dysfunction suggests the involvement of oxidative stress in mitochondrial dysfunction. For the role of NAC in mitochondrial length, density and morphology in EH-treated cells, NAC efficiently attenuated EH-induced morphological alterations of the mitochondria, which were corroborated by increased mitochondrial length and density as shown in Fig. 6N–Q. These results indicated that the antioxidant effect of NAC played neuroprotective role in EH induced cell injury by inhibiting ROS production, mitochondrial functional and structural impairments, which was similar to the effect of rGDF11 treatment.

### Treatment with mitochondrial division inhibitor Mdivi-1 rescues erythrocyte homogenate induced apoptosis and mitochondrial dysfunction in SH-SY5Y cells

**Figure 7:**
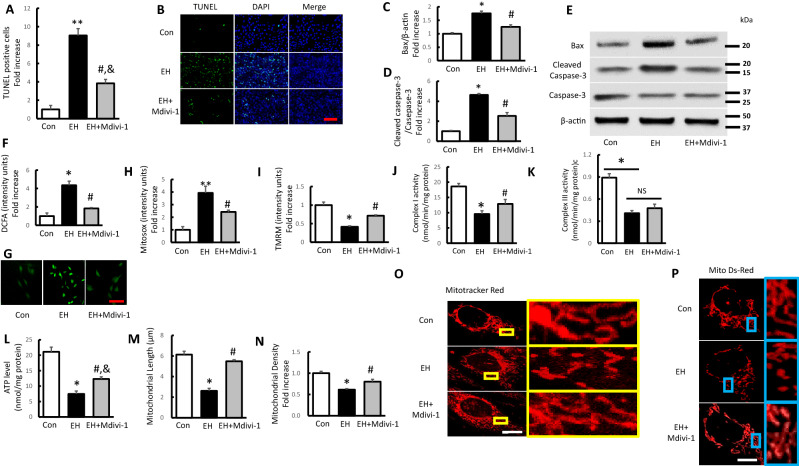
Treatment with mitochondrial division inhibitor Mdivi-1 rescues EH-induced apoptosis and mitochondrial dysfunction in SH-SY5Y cells. (**A**,**B**) The TUNEL-positive cells presenting as fold increase and representative pictures for TUNEL/DAPI-fluorescence staining (scale bar 200 μm) in indicated groups. (**C**–**E**) Quantification of immunoreactive bands for Bax relative to β-actin (**C**) and cleaved Caspase-3 relative to Caspase-3 (**D**). Immunoreactive bands for Bax, cleaved Caspase-3, and Caspase-3 in the different treated cells (**E**). (**F**,**G**) Quantitative analysis of DCFA fluorescence intensity presenting as fold increase and representative images (scale bar 50 μm) in the indicated groups. (**H**,**I**) Quantification of TMRM, Mitosox staining intensity presenting as fold increases in the indicated groups. (**J**–**L**) Complex I, III activities and ATP levels in the indicated groups. (**M**–**P**) Quantification of mitochondrial length and density with representative images of Mitotracker Red and Mito Ds-Red staining (scale bar 5 μm) in the indicated groups. Values shown as mean ± SD. *p < 0.05 and **p < 0.01 versus control group; ^#^p < 0.05 versus EH group and ^&^p < 0.05 versus control group (n = 7).

Over-fission of mitochondria plays an important role on mitochondrial and neuronal dysfunction under oxidative circumstance^37^. Results presented above raise the question of whether GDF11 rescued EH induced mitochondrial and neuronal damages via inhibiting mitochondrial division. To address the questions, we investigated the effect of mitochondrial division inhibitor, Mdivi-1, a selective inhibitor of GTPase activity in Drp1. Treatment with Mdivi-1 successfully attenuated cell apoptosis and expression of relative proteins (Fig. 7A–E). In addition, intracellular and mitochondrial ROS level induced by EH treatment were significantly inhibited when exposed to Mdivi-1 (Fig. 7F–H). Furtherly, we tested mitochondrial membrane potential, respiration chain activity and ATP production following treatment with Mdivi-1. Despite of no statistical difference found in complex III activity, deficits in mitochondrial membrane potential, complex I activity and ATP level were significantly reversed by Mdivi-1 intervention (Fig. 7I–L). Moreover, the loss of mitochondrial length and density associated with mitochondrial fragmentation induced by EH was significantly eradicated with the treatment with Mdivi-1 (Fig. 7M–P). Collectively, our data indicated that Mdivi-1 played neuroprotective role in EH induced cell injury by inhibiting mitochondrial over-fission, leading to attenuated ROS production, and improved mitochondrial function, and GDF11 might rescue EH-induced mitochondrial and neuronal damages via inhibiting mitochondrial division.

## Discussion

Consensus has already been made that ICH-induced oxidative stress (OS) is one major mechanism involved in subsequent damages of ICH such as brain edema, inflammatory recruitment, neuronal apoptosis as well as mitochondrial toxicity^1,3,6^. As the main component of hematoma, red blood cell(RBC) lyses into hemoglobin (Hb), which itself could release a large amount of superoxide during spontaneous nonenzymatic oxidation to oxyhemoglobin and methemoglobin^52,53^, triggers downstream ROS production. Though the specific pathways are still unclear, NADPH Oxidase(NOX) or nitric oxide synthase(NOS) activated by Hb may possibly involve in ROS mediation^54,55^. The anti-aging and anti-oxidative stress effect of GDF11 has been proved in multiple systems, moreover the neuroprotective potential of GDF11 on ICH was initially revealed in our previous study^1^. However, the molecular mechanisms linking ROS accumulation to mitochondrial deficit after ICH remains veiled. In our study, we, for the first time, explored the protective role of GDF11 on post-ICH secondary injuries by attenuating ROS-mediated mitochondrial dynamic abnormality and dysfunction.

In vivo, GDF11 could significantly improve the neurological deficits and peri-hematomal edema after ICH. Our study also found that ICH-induced neuron apoptosis and inflammation were remarkably down-regulated by GDF11. The inherent connection of pathological apoptosis and inflammatory reaction to ICH-induced brain edema and neuro-functional disability has been deeply clarified in previous studies^6^. For anti-apoptosis and anti-inflammation effects of GDF11, the positive findings were documented in multiple systems^26–28,56^. Zhang et al. reported that apoptosis of endothelial progenitor cells could be attenuated by GDF11 in diabetic limb ischemia^57^. On cardiovascular study, GDF11 could decrease endothelial apoptosis and inflammation against atherosclerosis in aging mice^58^. Moreover, in neurologic system, the neuro-inflammatory activity was significantly decreased in Alzheimer’s disease model after GDF11 treatment^59^. In consistency, those potentials of GDF11 on ICH were again confirmed in our study. At upstream, ROS generation plays an essential role on inducting inflammatory mediators and activating neuron death. In this experiment, the depression of OS status explained upstream regulation of GDF11 on ICH secondary damages.

Mitochondria, vital organelles maintaining the intracellular energy homeostasis and function, are the site of intracellular oxygen free radical production as well as the main target of oxygen free radicals^60^. Given the critical role of mitochondria in neurons, mitochondrial dysfunction and morphology deformation are increasingly implicated in OS injuries^61^. Mitochondrial membrane potential (MMP), formed by a proton gradient established across the mitochondrial inner membrane, is a vital index of mitochondrial ATP production^61^. Some researchers explored exponential dependence of ROS production on MMP in respiratory stage 4 from rat heart muscle^62^. However, under the exposure of erythrocyte lysate, neuronal MMP was found declined associated with the boost of mtROS production, which in turn deteriorates oxidative damage^12^. In our study, also, MMP loss was tested via TMRM examination in EH-treated cells, moreover ATP decrease and ROS promotion were observed both in vivo and in vitro. While after GDF11 treatment, the collapse of MMP and increase of ROS were inhibited associated with rescue of ATP production, indicating the potential of GDF11 saving mitochondrial function against OS.

Oxidative phosphorylation of mitochondrial ETC plays an essential role on MMP homeostasis and ATP generation^63^. Activities of mitochondrial respiratory Complexes, including Complexes I, II, III, IV and V, must be very well orchestrated to function properly, otherwise subsequent oxidative damage and apoptosis could be caused by the respiratory chain incoordination^64^. Post-ICH OS injury and neuron apoptosis were highlighted in previous articles, but the evidence on mitochondrial respiratory chain’s obstacle in ICH-induced apoptosis remains scarce. Within electron transfer and mitochondrial metabolism links, the main process producing mitochondrial ROS exists on involvement of Complexes I and III^65^. According to previous research, Complex I and III could release superoxide towards the mitochondrial matrix and inner mitochondrial membrane, and defect of Complex I and III activity played a crucial role in mitochondrial OS, which contributes to apoptosis induction^65^. Our study identified that ICH suppressed activity of Complex I and III among the ETC enzymes consistent with down-regulation of ATP level. In in vivo and in vitro ICH models, GDF11 showed its protective effect on maintaining the activity of ETC complex I and III. However, the precise mechanisms underlying improvement in oxidative stress-related mitochondrial dysfunction are still not well understood.

Of note, impaired mitochondrial dynamics are deeply involved in the pathophysiological process of neuro-damages. Our results showed that erythrocyte homogenate could induce the imbalance of mitochondrial fission, EH treatment apparently resulted in more mitochondrial fragmentation in neurons, indicated as decreased mito-density and shortened mito-length. And morphological appearance displayed fragmented, misshapen, bleb-like mitochondria. EH treatment induced excessive mitochondrial fragmentation could be alleviated by GDF11. Compared to EH induced cohort, improvements of mitochondrial quantity and morphology were shown in cells after GDF11 treatment, supporting the regulative potential of GDF11 on the mitochondrial dynamics. Drp1 and Fis1 are important proteins leading to mitochondrial fission, while Mfn2 and Opa1 promotes the fusion^22,66^. And Drp1 phosphorylation at 616 residues is closely associated with mitochondrial fission^49^. Our data demonstrated that GDF11 significantly suppressed the up-regulation of Drp1, p-Drp1 (at 616 residues), as well as p-Drp1 contrast with total Drp1 protein levels in EH treated cells, presented no obvious change in Mfn2 and Opa1 expression.

Given that the imbalance of mitochondrial fission and fusion plays a critical role in maintenance of mitochondrial morphology, distribution and function^48^, we assessed the link between oxidative stress and changes in mitochondrial dynamics. Previous studies reported that the process of mitochondrial fission and fragmentation is mediated by Drp1, which could be upregulated by ROS production^9^. Thus, we hypothesized that inhibited ROS production or Drp1 activity could also attenuate the aforementioned neuronal and mitochondrial damage indicators. Therefore, we further tested the role of NAC and Mdivi-1 on cells with EH treatment. Consistent with the prediction, similarly to GDF11, NAC and Mdivi-1 treatment respectively rescued apoptosis and mitochondrial dysfunctions by reversing the inner MMP, mitochondrial ROS production/accumulation, ETC activities and morphological change. Recent evidence indicated that aberrant mitochondrial morphology may enhance ROS formation, which, in turn, may deteriorate mitochondrial ultrastructure^67^. Our findings suggested that GDF 11 may exert neuroprotective effect against oxidative stress by inhibiting mitochondrial functional and structural pathological damage after ICH. GDF11, a member of TGF-β super-family, acts through the activin type II receptor (ActRIIB) and mediates downstream signaling through the activation of the SMAD2/SMAD3 complex^25^. Previous studies on myocardial ischaemia/reperfusion injury also demonstrated that GDF11 administration increased the phosphorylation of SMAD3 and decreased the phosphorylation of FOXO3a, which then affected mitochondrial Ca^2+^ homeostasis, ROS accumulation and mitochondrial depolarization^26,29^. We suggested TGF-β1-Smad2/Smad3 signaling might underlie the neuroprotective effects of GDF11 in ICH, however, further studies are needed to better elucidate the pathway.

**Figure 8:**
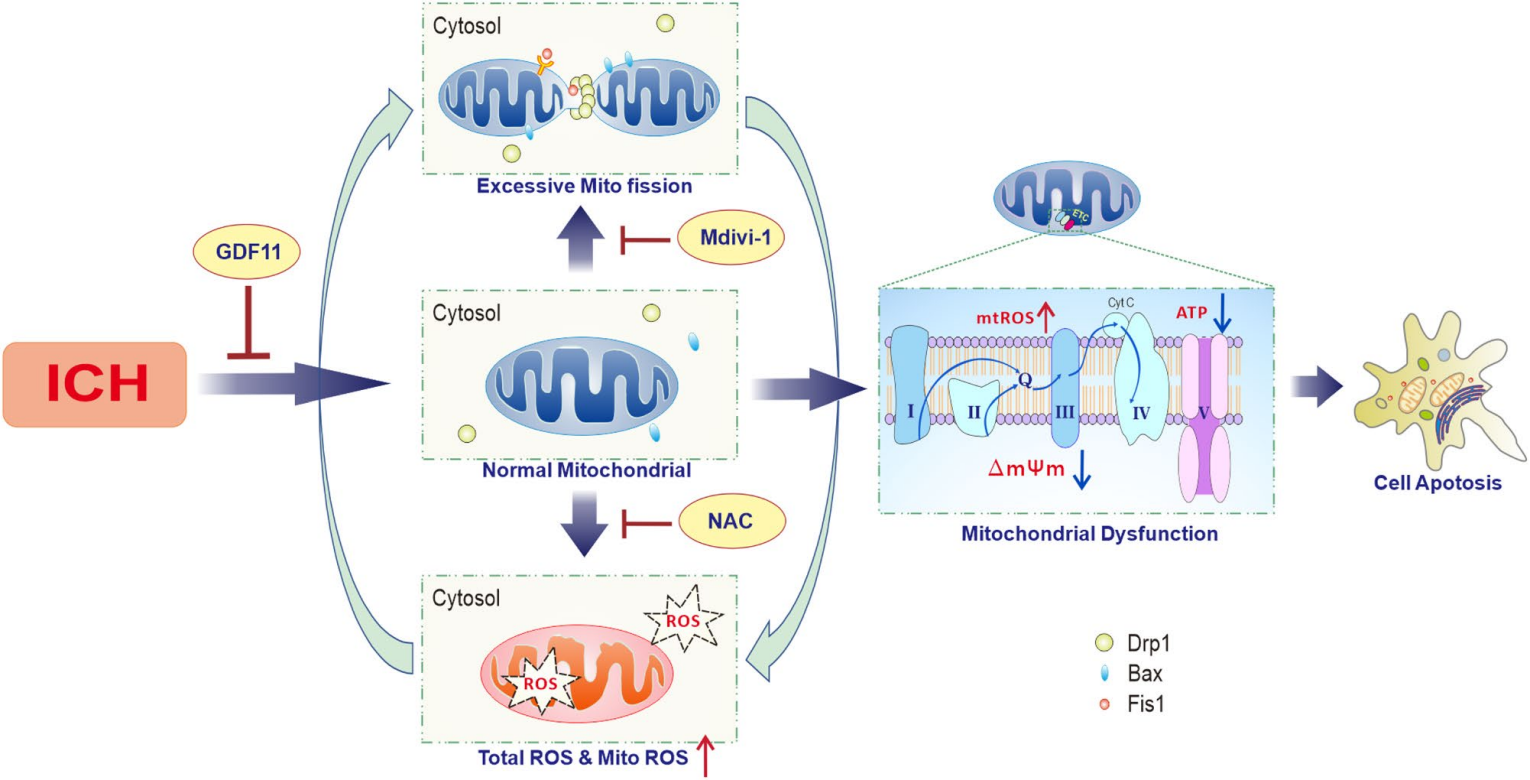
Working hypothesis: GDF11 protected the post-ICH secondary injury by suppressing the feedback loop between mitochondrial ROS production and mitochondrial dynamic alteration, resulting in attenuated mitochondrial function, consequently restoring cell apoptosis, and amelioration of neural damage.

Taken together, our study offered new insights into the underlying mechanisms of the GDF11 neuroprotection on ICH pathogenesis. For the first time, we proposed that GDF11 protected the post-ICH secondary injury by attenuating ROS-mediated mitochondrial dynamic abnormality and dysfunction, resulting in attenuated cell apoptosis, and amelioration of neural damage (Fig. 8). These studies suggest therapeutic potential of GDF11 in treating ICH-induced injuries and explore targets for the development of interventions to prevent or treat ICH-induced mitochondrial deficits.

## Supplementary Information

Supplementary Information 1.

## References

[1] Anqi X, Ruiqi C, Yanming R, Chao Y (2019). Neuroprotective potential of GDF11 in experimental intracerebral hemorrhage in elderly rats. J. Clin. Neurosci..

[2] Liu R (2017). CD163 expression in neurons after experimental intracerebral hemorrhage. Stroke.

[3] Bhat AH (2015). Oxidative stress, mitochondrial dysfunction and neurodegenerative diseases; a mechanistic insight. Biomed. Pharmacother..

[4] Qu J, Chen W, Hu R, Feng H (2016). The injury and therapy of reactive oxygen species in intracerebral hemorrhage looking at mitochondria. Oxid. Med. Cell. Longev..

[5] Shi E (2019). Chronic inflammation, cognitive impairment, and distal brain region alteration following intracerebral hemorrhage. FASEB J..

[6] Hu X (2016). Oxidative stress in intracerebral hemorrhage: Sources, mechanisms, and therapeutic targets. Oxid. Med. Cell. Longev..

[7] Zhao X (2009). Neuroprotective role of haptoglobin after intracerebral hemorrhage. J. Neurosci..

[8] Zia MT, Ungvari Z, Csiszar A, Ballabh P (2008). Free radical generation in germinal matrix hemorrhage. FASEB J..

[9] Xiao A (2017). The cyclophilin D/Drp1 axis regulates mitochondrial fission contributing to oxidative stress-induced mitochondrial dysfunctions in SH-SY5Y cells. Biochem. Biophys. Res. Commun..

[10] Brunswick AS (2012). Serum biomarkers of spontaneous intracerebral hemorrhage induced secondary brain injury. J. Neurol. Sci..

[11] Kim-Han JS, Kopp SJ, Dugan LL, Diringer MN (2006). Perihematomal mitochondrial dysfunction after intracerebral hemorrhage. Stroke.

[12] Zheng J (2018). Sirt3 ameliorates oxidative stress and mitochondrial dysfunction after intracerebral hemorrhage in diabetic rats. Front. Neurosci..

[13] Qu X (2019). RNF34 overexpression exacerbates neurological deficits and brain injury in a mouse model of intracerebral hemorrhage by potentiating mitochondrial dysfunction-mediated oxidative stress. Sci. Rep..

[14] Nuttall A, Foster S, Zhang Y, Wilson T (2015). Mitochondria dynamics in auditory cells. FASEB J..

[15] Smirnova E, Griparic L, Shurland DL, van der Bliek AM (2001). Dynamin-related protein Drp1 is required for mitochondrial division in mammalian cells. Mol. Biol. Cell..

[16] Chen H (2003). Mitofusins Mfn1 and Mfn2 coordinately regulate mitochondrial fusion and are essential for embryonic development. J. Cell. Biol..

[17] Detmer SA, Chan DC (2007). Functions and dysfunctions of mitochondrial dynamics. Nat. Rev. Mol. Cell. Biol..

[18] Song Z, Ghochani M, McCaffery JM, Frey TG, Chan DC (2009). Mitofusins and OPA1 mediate sequential steps in mitochondrial membrane fusion. Mol. Biol. Cell..

[19] Hollenbeck PJ, Saxton WM (2005). The axonal transport of mitochondria. J. Cell. Sci..

[20] Chen H, McCaffery JM, Chan DC (2007). Mitochondrial fusion protects against neurodegeneration in the cerebellum. Cell.

[21] Verstreken P (2005). Synaptic mitochondria are critical for mobilization of reserve pool vesicles at *Drosophila neuromuscular* junctions. Neuron.

[22] Burte F, Carelli V, Chinnery PF, Yu-Wai-Man P (2015). Disturbed mitochondrial dynamics and neurodegenerative disorders. Nat. Rev. Neurol..

[23] Patel K, Amthor H (2005). The function of Myostatin and strategies of Myostatin blockade-new hope for therapies aimed at promoting growth of skeletal muscle. Neuromuscul. Disord..

[24] Lee SJ, McPherron AC (2001). Regulation of myostatin activity and muscle growth. Proc. Natl. Acad. Sci. USA.

[25] Rochette L, Zeller M, Cottin Y, Vergely C (2015). Growth and differentiation factor 11 (GDF11): Functions in the regulation of erythropoiesis and cardiac regeneration. Pharmacol. Ther..

[26] Leinwand LA, Harrison BC (2013). Young at heart. Cell.

[27] Andersen RE, Lim DA (2014). An ingredient for the elixir of youth. Cell. Res..

[28] Katsimpardi L (2014). Vascular and neurogenic rejuvenation of the aging mouse brain by young systemic factors. Science.

[29] Yang Y (2017). Does growth differentiation factor 11 protect against myocardial ischaemia/reperfusion injury? A hypothesis. J. Int. Med. Res..

[30] Zhao Y (2020). The neuroprotective and neurorestorative effects of growth differentiation factor 11 in cerebral ischemic injury. Brain Res..

[31] Camici GG, Savarese G, Akhmedov A, Luscher TF (2015). Molecular mechanism of endothelial and vascular aging: Implications for cardiovascular disease. Eur. Heart J..

[32] Wang Z (2018). Melatonin alleviates intracerebral hemorrhage-induced secondary brain injury in rats via suppressing apoptosis, inflammation, oxidative stress, DNA damage, and mitochondria injury. Transl. Stroke Res..

[33] He P (2008). PBDE-47-induced oxidative stress, DNA damage and apoptosis in primary cultured rat hippocampal neurons. Neurotoxicology.

[34] Rotruck JT (1973). Selenium: Biochemical role as a component of glutathione peroxidase. Science.

[35] Habig WH, Pabst MJ, Jakoby WB (1974). Glutathione S-transferases. The first enzymatic step in mercapturic acid formation. J. Biol. Chem..

[36] Tieu K (2003). D-beta-hydroxybutyrate rescues mitochondrial respiration and mitigates features of Parkinson disease. J. Clin. Invest..

[37] Gan X (2014). Inhibition of ERK-DLP1 signaling and mitochondrial division alleviates mitochondrial dysfunction in Alzheimer's disease cybrid cell. Biochim. Biophys. Acta.

[38] Birch-Machin MA, Turnbull DM (2001). Assaying mitochondrial respiratory complex activity in mitochondria isolated from human cells and tissues. Methods Cell. Biol..

[39] Du H (2008). Cyclophilin D deficiency attenuates mitochondrial and neuronal perturbation and ameliorates learning and memory in Alzheimer's disease. Nat. Med..

[40] Shi Y, Liu JP (2011). Gdf11 facilitates temporal progression of neurogenesis in the developing spinal cord. J. Neurosci..

[41] Zhao X (2007). Hematoma resolution as a target for intracerebral hemorrhage treatment: Role for peroxisome proliferator-activated receptor gamma in microglia/macrophages. Ann. Neurol..

[42] Wu S, Zhou F, Zhang Z, Xing D (2011). Mitochondrial oxidative stress causes mitochondrial fragmentation via differential modulation of mitochondrial fission–fusion proteins. FEBS J..

[43] Wang D (2017). Anti-high mobility group box-1 (HMGB1) antibody inhibits hemorrhage-induced brain injury and improved neurological deficits in rats. Sci. Rep..

[44] Shin DH, Kim OH, Jun HS, Kang MK (2008). Inhibitory effect of capsaicin on B16–F10 melanoma cell migration via the phosphatidylinositol 3-kinase/Akt/Rac1 signal pathway. Exp. Mol. Med..

[45] Gan X, Huang S, Yu Q, Yu H, Yan SS (2015). Blockade of Drp1 rescues oxidative stress-induced osteoblast dysfunction. Biochem. Biophys. Res. Commun..

[46] Wagner KR, Sharp FR, Ardizzone TD, Lu A, Clark JF (2003). Heme and iron metabolism: Role in cerebral hemorrhage. J. Cereb. Blood Flow Metab..

[47] Thiex R, Tsirka SE (2007). Brain edema after intracerebral hemorrhage: Mechanisms, treatment options, management strategies, and operative indications. Neurosurg. Focus.

[48] Wai T, Langer T (2016). Mitochondrial dynamics and metabolic regulation. Trends Endocrinol. Metab..

[49] Kashatus DF (2011). RALA and RALBP1 regulate mitochondrial fission at mitosis. Nat. Cell. Biol..

[50] Prins JM, Brooks DM, Thompson CM, Lurie DI (2010). Chronic low-level Pb exposure during development decreases the expression of the voltage-dependent anion channel in auditory neurons of the brainstem. Neurotoxicology.

[51] Wang S, Chi Q, Hu X, Cong Y, Li S (2019). Hydrogen sulfide-induced oxidative stress leads to excessive mitochondrial fission to activate apoptosis in broiler myocardia. Ecotoxicol. Environ Saf..

[52] Misra HP, Fridovich I (1972). The generation of superoxide radical during the autoxidation of hemoglobin. J. Biol. Chem..

[53] Huang FP (2002). Brain edema after experimental intracerebral hemorrhage: Role of hemoglobin degradation products. J. Neurosurg..

[54] Tang J (2005). Role of NADPH oxidase in the brain injury of intracerebral hemorrhage. J. Neurochem..

[55] Laird MD, Wakade C, Alleyne CH, Dhandapani KM (2008). Hemin-induced necroptosis involves glutathione depletion in mouse astrocytes. Free Radic. Biol. Med..

[56] Brun CE, Rudnicki MA (2015). GDF11 and the mythical fountain of youth. Cell Metab..

[57] Zhang J (2018). GDF11 improves angiogenic function of EPCs in diabetic limb ischemia. Diabetes.

[58] Mei W (2016). GDF11 protects against endothelial injury and reduces atherosclerotic lesion formation in apolipoprotein E-null mice. Mol. Ther..

[59] Zhang W (2018). GDF11 rejuvenates cerebrovascular structure and function in an animal model of Alzheimer's disease. J. Alzheimers Dis..

[60] Zhang L (2016). Reactive oxygen species regulatory mechanisms associated with rapid response of MC3T3-E1 cells for vibration stress. Biochem. Biophys. Res. Commun..

[61] Sb H (2019). The vicious circle between mitochondrial oxidative stress and dynamic abnormality mediates triethylene glycol dimethacrylate-induced preodontoblast apoptosis. Free Radic. Biol. Med..

[62] Korshunov SS, Skulachev VP, Starkov AA (1997). High protonic potential actuates a mechanism of production of reactive oxygen species in mitochondria. FEBS Lett..

[63] Feichtinger RG (2014). Alterations of oxidative phosphorylation complexes in astrocytomas. Glia.

[64] Drose S, Brandt U, Wittig I (2014). Mitochondrial respiratory chain complexes as sources and targets of thiol-based redox-regulation. Biochim. Biophys. Acta.

[65] Fan L (2017). MicroRNA-661 enhances TRAIL or STS induced osteosarcoma cell apoptosis by modulating the expression of cytochrome c1. Cell. Physiol. Biochem..

[66] Mishra P, Chan DC (2014). Mitochondrial dynamics and inheritance during cell division, development and disease. Nat. Rev. Mol. Cell. Biol..

[67] Jezek J, Cooper KF, Strich R (2018). Reactive oxygen species and mitochondrial dynamics: The Yin and Yang of mitochondrial dysfunction and cancer progression. Antioxidants (Basel).

