# Supplementary material for: GDF11 alleviates secondary brain injury after intracerebral hemorrhage via attenuating mitochondrial dynamic abnormality and dysfunction
Source: Sci Rep. 2021 Feb 17;11:3974. doi: 10.1038/s41598-021-83545-x (PMC7889617; doi:10.1038/s41598-021-83545-x)
Supplement: Supplementary file 1 — Supplementary Information 1. [file 41598_2021_83545_MOESM1_ESM.pdf]

Figure 3J

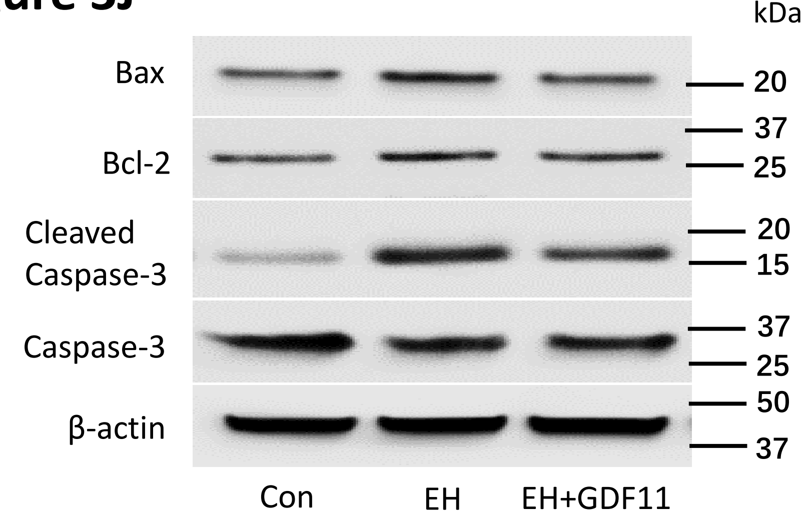

Original band for Figure 3J:

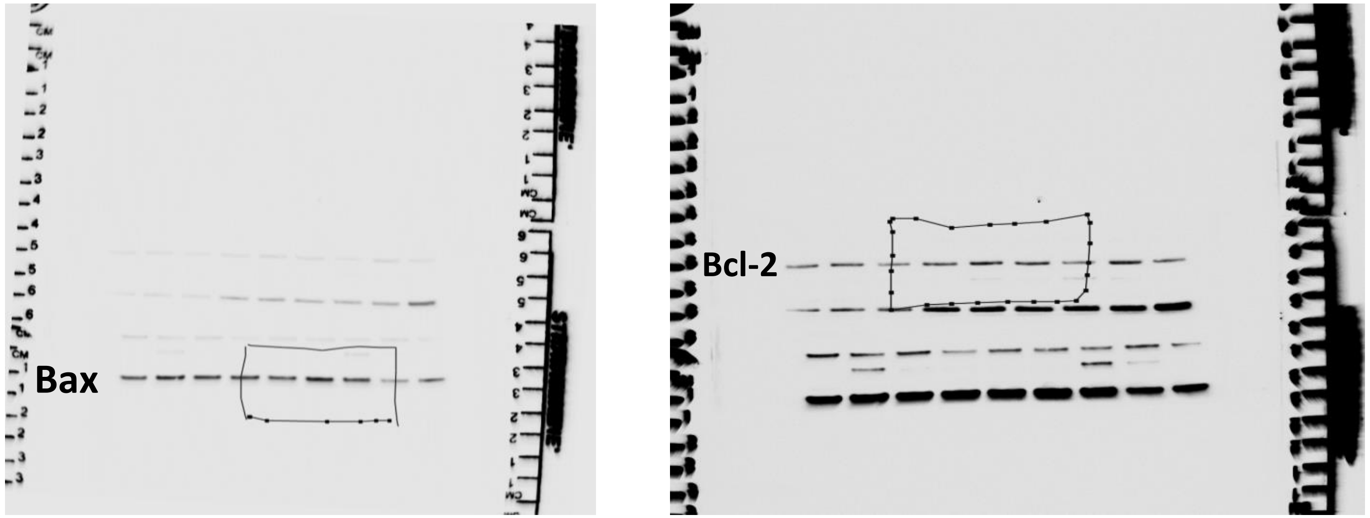

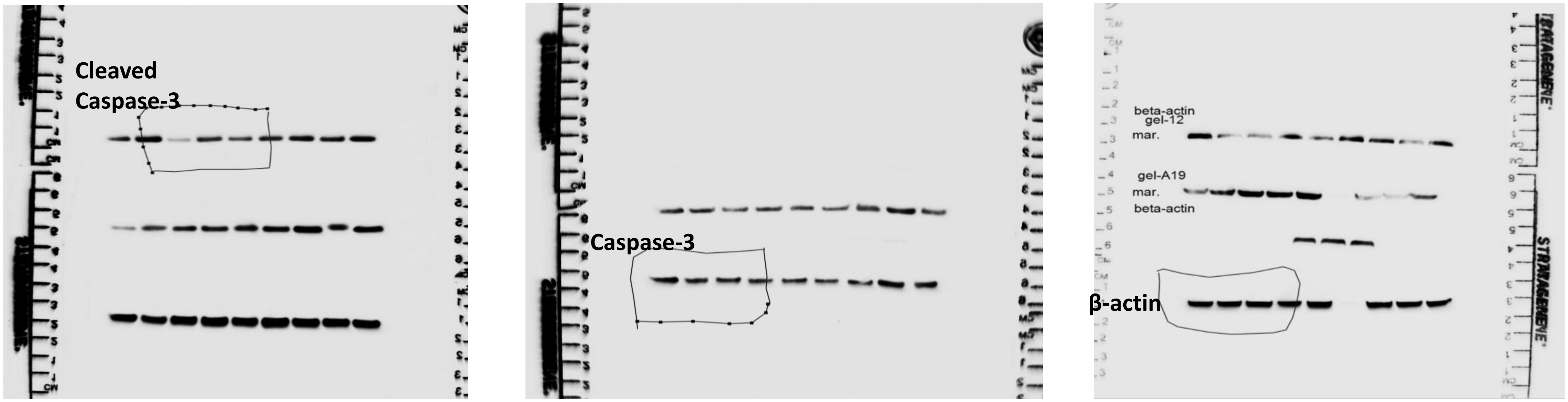

Figure S1

Figure 5L

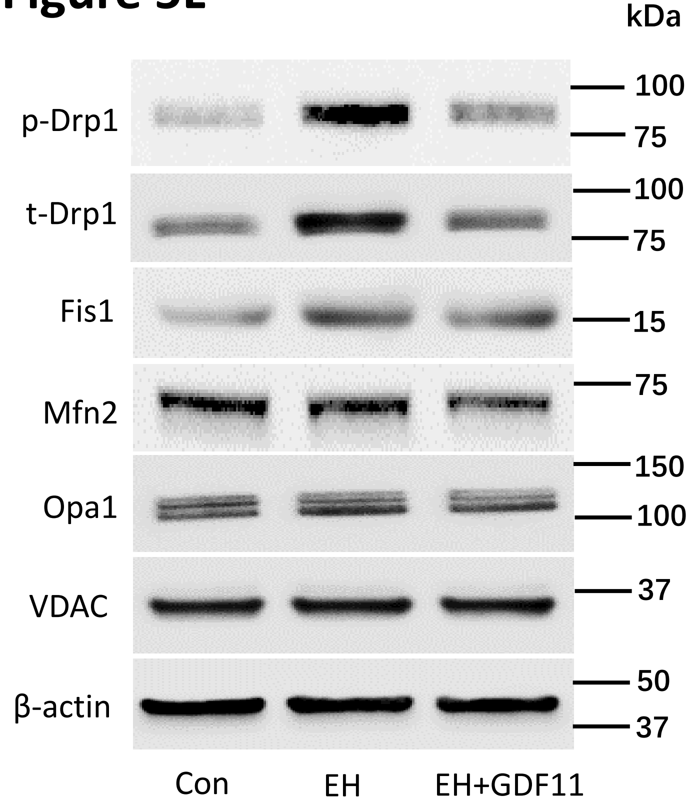

Original band for Figure 5L:

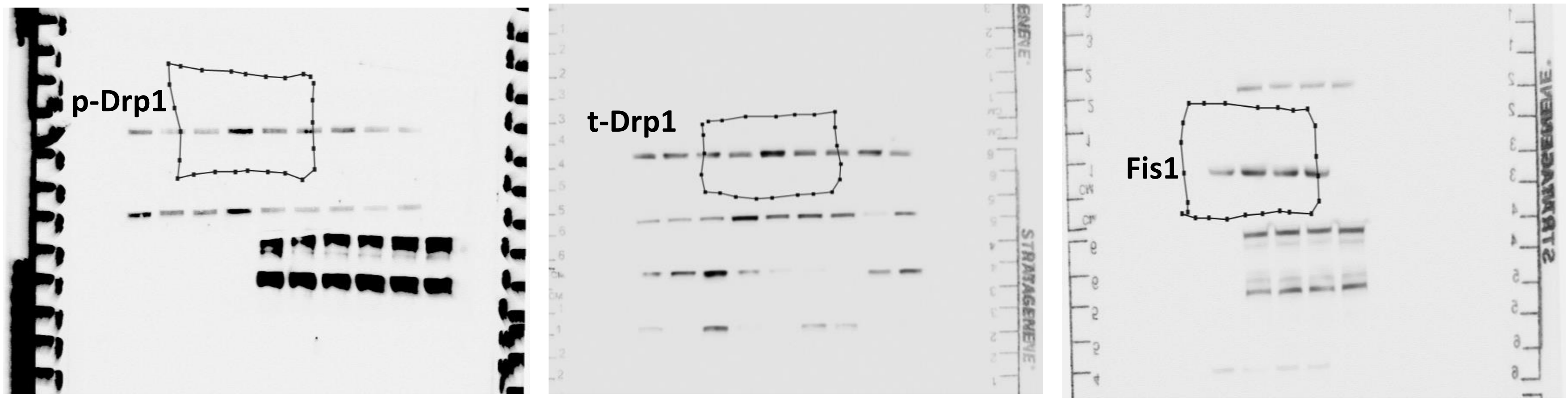

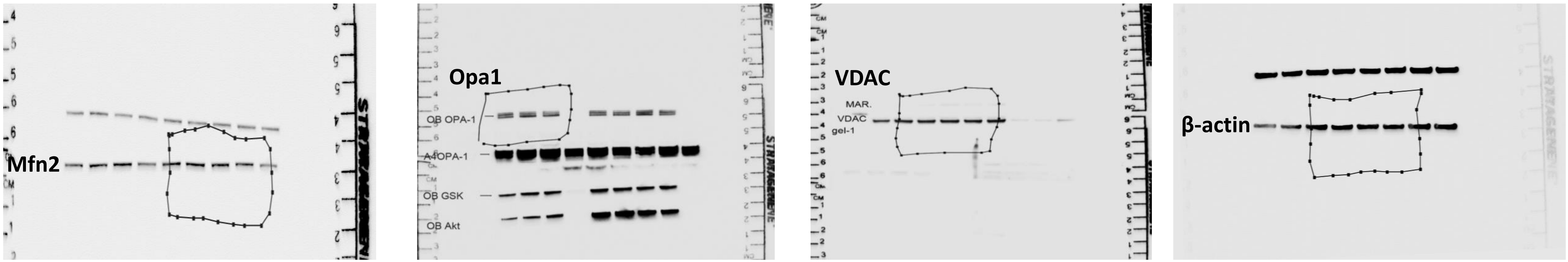

Figure S2

Figure 6E

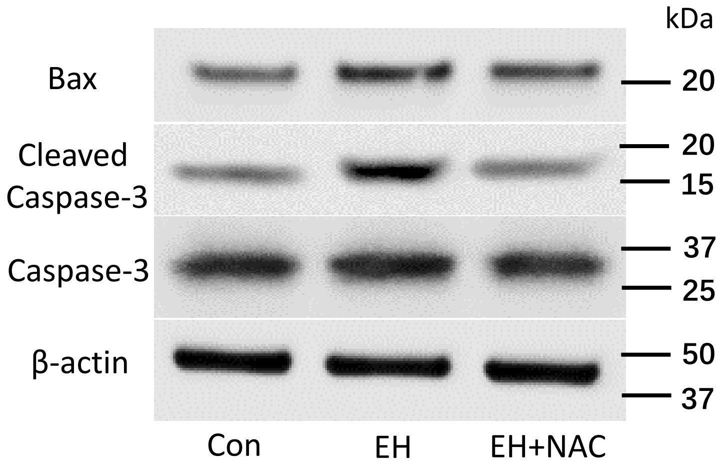

Original band for Figure 6E:

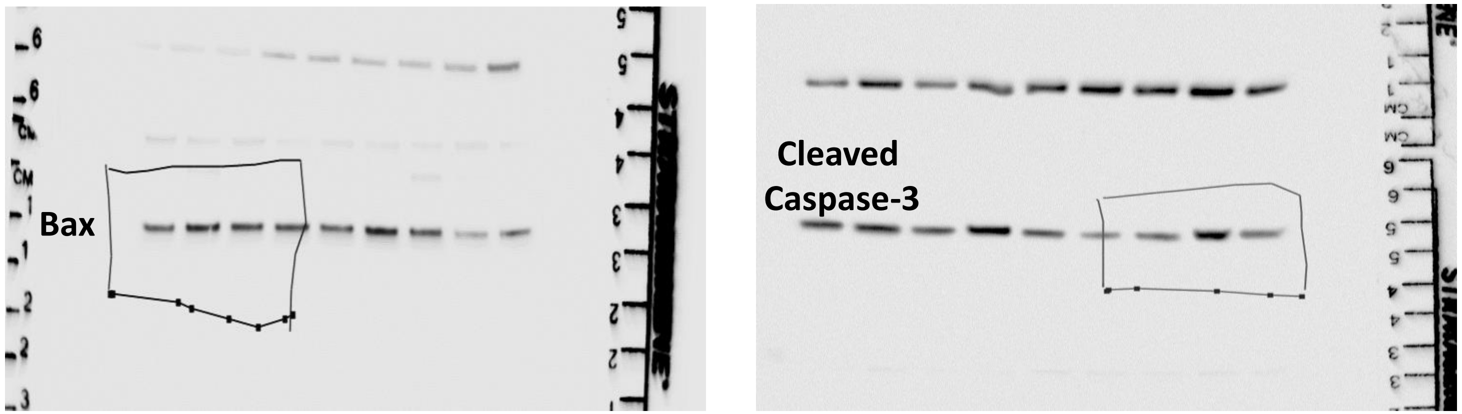

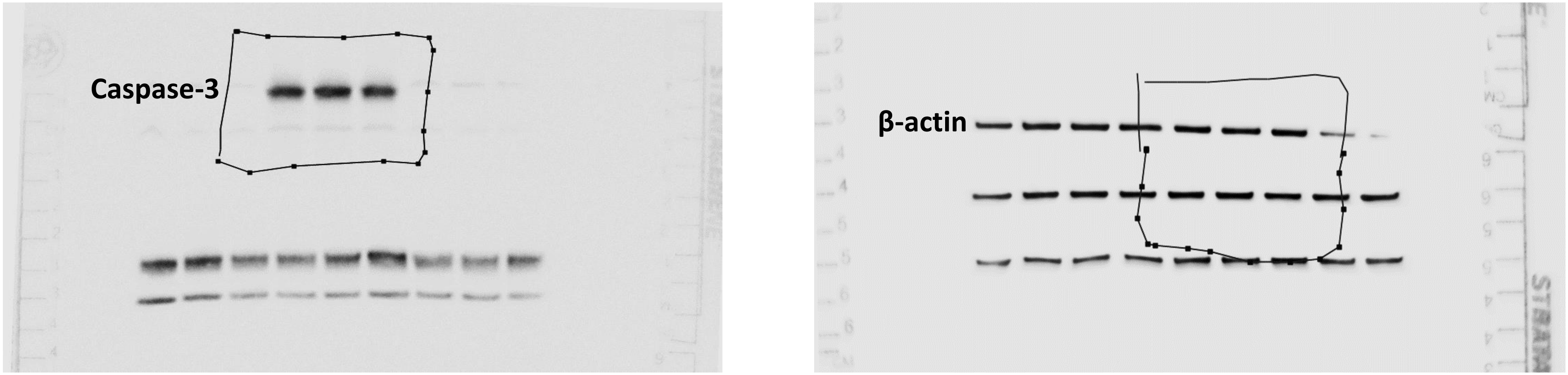

Figure S3

Figure 7E

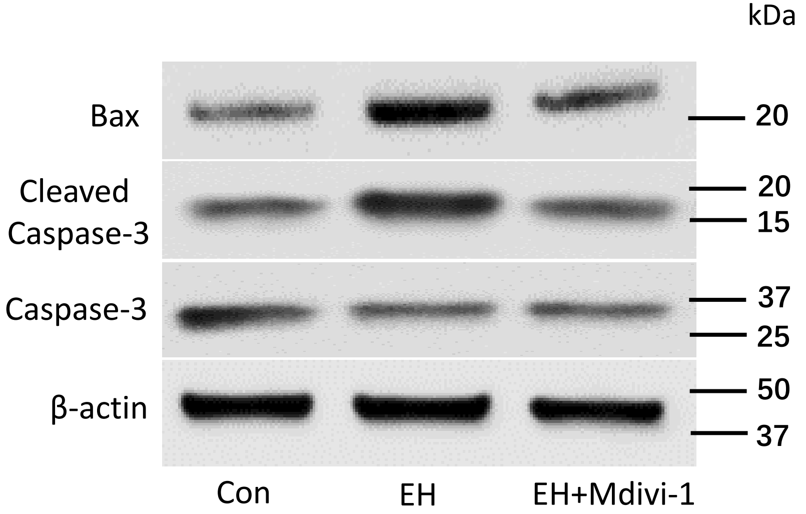

Original band for Figure 7E:

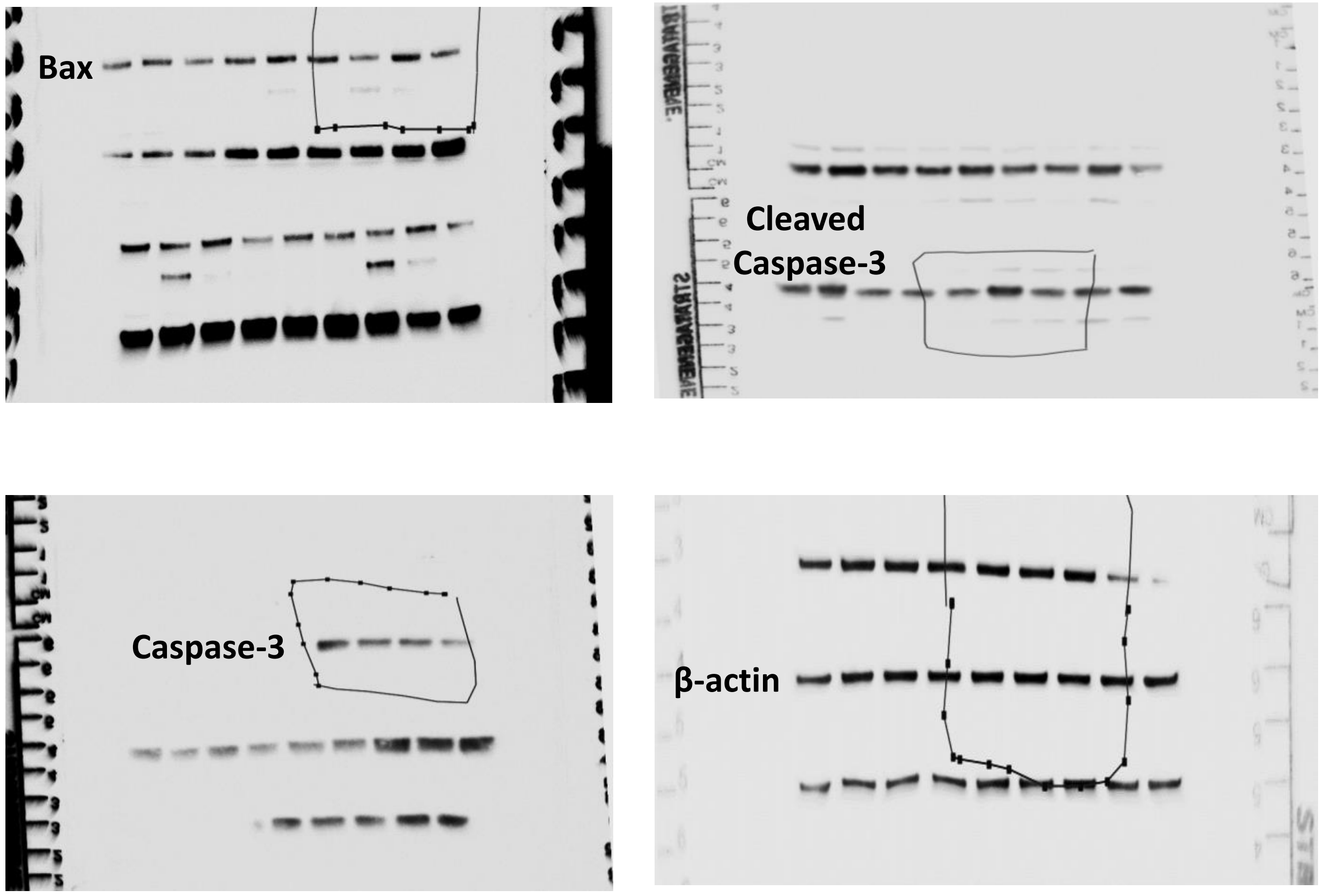

Figure S4
